# Involvment of Cytosolic and Mitochondrial GSK-3β in Mitochondrial Dysfunction and Neuronal Cell Death of MPTP/MPP^+^-Treated Neurons

**DOI:** 10.1371/journal.pone.0005491

**Published:** 2009-05-11

**Authors:** Agnès Petit-Paitel, Frédéric Brau, Julie Cazareth, Joëlle Chabry

**Affiliations:** Institut de Pharmacologie Moléculaire et Cellulaire, Centre National de la Recherche Scientifique, Unité Mixte de Recherche 6097, Valbonne, France; Health Canada, Canada

## Abstract

Aberrant mitochondrial function appears to play a central role in dopaminergic neuronal loss in Parkinson's disease (PD). 1-methyl-4-phenylpyridinium iodide (MPP^+^), the active metabolite of *N*-methyl-4-phenyl-1,2,3,6-tetrahydropyridine (MPTP), is a selective inhibitor of mitochondrial complex I and is widely used in rodent and cell models to elicit neurochemical alterations associated with PD. Recent findings suggest that Glycogen Synthase Kinase-3β (GSK-3β), a critical activator of neuronal apoptosis, is involved in the dopaminergic cell death. In this study, the role of GSK-3β in modulating MPP^+^-induced mitochondrial dysfunction and neuronal death was examined *in vivo*, and in two neuronal cell models namely primary cultured and immortalized neurons. In both cell models, MPTP/MPP^+^ treatment caused cell death associated with time- and concentration-dependent activation of GSK-3β, evidenced by the increased level of the active form of the kinase, i.e. GSK-3β phosphorylated at tyrosine 216 residue. Using immunocytochemistry and subcellular fractionation techniques, we showed that GSK-3β partially localized within mitochondria in both neuronal cell models. Moreover, MPP^+^ treatment induced a significant decrease of the specific phospho-Tyr216-GSK-3β labeling in mitochondria concomitantly with an increase into the cytosol. Using two distinct fluorescent probes, we showed that MPP^+^ induced cell death through the depolarization of mitochondrial membrane potential. Inhibition of GSK-3β activity using well-characterized inhibitors, LiCl and kenpaullone, and RNA interference, prevented MPP^+^-induced cell death by blocking mitochondrial membrane potential changes and subsequent caspase-9 and -3 activation. These results indicate that GSK-3β is a critical mediator of MPTP/MPP^+^-induced neurotoxicity through its ability to regulate mitochondrial functions. Inhibition of GSK-3β activity might provide protection against mitochondrial stress-induced cell death.

## Introduction

Parkinson's disease (PD) is the second most common neurological disorder characterized by motor and behavioral disturbances caused by the degeneration of dopaminergic neurons in the *substantia nigra pars compacta* (SNc) [1]. The molecular events underlying the loss of dopaminergic neurons in PD remain unclear, although substantial evidence suggest that mitochondrial dysfunction might be a major contributor [2], [3].

Two of the most important functions of mitochondria relate to the production of ATP by oxidative phosphorylation and the mediation of signals for apoptotic cell death, the latter being particularly important in the context of PD. A defect in mitochondrial complex I activity in PD was identified in substantia nigra [2] as well as in platelet mitochondria of PD patients [4].

Experimentally, inhibitors of complex I of the mitochondrial respiratory chain can produce the selective neuronal loss and consequent behavioral deficits mimicking PD hallmarks. For instance, in rodents, human and non-human primates, the neurotoxin 1-methyl-1,2,3,6-tetrahydropyridine (MPTP) mediates selective damage to dopaminergic neurons of the nigro-striatal pathway [5]. Neuronal cell death is caused by MPP^+^, the active metabolite of MPTP, which induces oxidative stress and the opening of the mitochondrial permeability transition pore (mPTP), the release of cytochrome c and the activation of caspases [6]–[8]. The mPTP results in increased permeability of the inner mitochondrial membrane to protons, ions and other solutes resulting in a decrease of the mitochondrial membrane potential (Δψm).

Glycogen Synthase Kinase (GSK)-3 is a proline-directed serine/threonine kinase originally identified as a regulator of glycogen synthase [9]. Gradually, GSK-3 appeared to be a multifaceted enzyme, affecting a wide range of biological functions including gene expression, cellular architecture and apoptosis [10]. Of the two closely related isoforms, GSK-3α and GSK-3β, GSK-3β is known to play critical roles in oxidative stress-induced neuronal apoptosis and pathogenesis of neurodegenerative diseases [11]–[17]. GSK-3β is activated by phosphorylation of the tyrosine 216 residue (Tyr216) located in the kinase domain and inactivated by phosphorylation of the amino-terminal serine 9 residue (Ser9) [18].

Recently, it has been shown that GSK-3β inhibition protects dopaminergic neurons from MPTP toxicity, suggesting that GSK-3β may play a key role in PD pathogenesis [19], [20]. Moreover, a chronic treatment with low lithium doses, a GSK-3β inhibitor, could induce neuroprotection in a mouse model of MPTP-induced striatal dopaminergic neurodegeneration and dopamine depletion [21]. Finally, two other PD mimetics, 6-hydroxydopamine (6-OHDA) and rotenone, induce neuronal death through the activation of GSK-3β [22], [23], [24].

The presence of GSK-3β within mitochondria has been reported [25]–[28]. Many reports indicate that the inhibition of GSK-3β reduces ischemia/reperfusion cardiac injury through the regulation of the mPTP opening, indicating that it could exert its functions by modulating mitochondrial activity [29]–[32].

The present study was designed to investigate the putative role of GSK-3β in MPTP/MPP^+^-induced mitochondrial dysfunction and dopaminergic neuronal death, *in vivo*, as well as in primary cultures of neuronal cells and immortalized neurons. Overall, MPTP/MPP^+^ induced the activation of GSK-3β by the phosphorylation of Tyr216 in mouse brain and in both neuronal cell models. However, on subcellular fractions of neurons, we established that MPP^+^ treatment induced specific activation of the cytosolic pool whereas it decreased the mitochondrial pool of active GSK-3β. This leads to the depolarization of the mitochondrial membrane, to the activation of caspase-9 and -3 and finally to neuronal cell death. Blockade of GSK-3β activity by selective inhibitors i.e. lithium or kenpaullone or siRNA-mediated GSK-3β knock-down efficiently abolished these effects. These findings suggest that GSK-3β activity is critical for neuronal death in response to the mitochondrial stressor MPTP/MPP^+^.

## Materials and Methods

### Ethics statements

Experimental procedures on mice and the care given to animals have been approved by the French Regional Committee for Animal Use and Care # NCA/2004/06-02.

### Animals

Seven weeks-old male C57BL/6 mice were purchased from Charles River Laboratories (L'arbresle, France). All mice were held in a temperature-controlled room maintained under a 12-h light/dark cycle and had access to food and water available *ad libitum*.

### *In vivo* MPTP treatment

Adult mice (n = 5/group) received one intraperitoneal (i.p.) injection of MPTP per day (30 mg of free base/kg of body weight per injection; Sigma-Aldrich, St Quentin Fallavier, France) for five consecutive days whereas control mice (n = 5/group) were treated in the same conditions with vehicle only (0.9% NaCl). Mice were sacrificed 1, 3, 5, and 7 days after the last injection. After decapitation, collected brains were quickly frozen in 2-methyl butane at −30°C and kept at −20°C until use. MPTP handling and safety measures were in accordance with published guidelines [33].

### Immunohistochemistry

Brain coronal sections (12 µm thickness) were performed on a cryostat (Leica, Nussloch, Germany). For double-staining experiments, rabbit anti-TH (anti-Tyrosine Hydroxylase, Cell Signaling, St Quentin-en-Yvelines, France) was used together with mouse anti-phospho-Tyr216-GSK-3β (BD Biosciences, Le Pont de Claix, France) as primary antibodies. Brain sections were permeabilized and blocked in PBS, pH 7.4, containing 0.2% CHAPS and 2% BSA (Sigma-Aldrich), then incubated with primary antibodies diluted in PBS, pH 7.4, containing 0.1% CHAPS and 2% BSA, for 3 h at room temperature. At the end of the incubation time, brain sections were washed three times with PBS and incubated with either Alexa488-conjugated or Texas-Red-conjugated secondary antibodies (Molecular Probes, Invitrogen, Cergy Pontoise, France) and diamidino-4′,6-phenylindol-2 dichlorhydrate (DAPI) for 1 h at room temperature. Sections were then washed three times with PBS and prepared for microscopy observation.

### Cell cultures

Murine TSM1 neuronal cells were grown in Opti-MEM (Invitrogen) supplemented with 10% inactivated fetal bovine serum (FBSi, BioWest, Nuaille, France), 100 U/mL penicillin, 100 mg/mL streptomycin, and 0.1 g/L G418 (Invitrogen).

Primary cultures of mouse neurons from embryonic day 13–14 C57BL/6 mice was carried out as described previously [34]. Briefly, neurons were cultured in Neurobasal medium containing B27 supplement (Invitrogen) and penicillin-streptomycin and used after 6–7 days of differentiation. Cell cultures were grown at 37°C in a humidified atmosphere of 5% C0_2_ and 95% air.

MPP^+^ (Sigma-Aldrich) was extemporaneously prepared by solubilization into appropriate cell culture medium. TSM1 and primary neuronal cell cultures were treated with the indicated concentrations of MPP^+^ for various times. Alternatively, neurons were pre-incubated for 15 min with indicated concentrations of LiCl or kenpaullone (kenp, Sigma-Aldrich) and then co-treated with 400 µM MPP^+^.

### siRNA cell transfection

GSK-3β-specific small interfering RNA (siRNA) or scrambled siRNA (Dharmacon, Perbio Science, Brebieres, France) were transfected into TSM1 cells using Oligofectamine (Invitrogen) according to the manufacturer's recommendations. Cells were used 48 h after transfection.

### Immunocytochemistry

TSM1 and primary cultures of neurons were grown on polylysine-coated glass coverslips. After treatment, cells were fixed with 4% paraformaldehyde then permeabilized in PBS pH 7.4, containing 0.2% CHAPS and 2% BSA. Cells were then incubated for 3 h at room temperature with the appropriate primary antibody (dilution 1/200) in PBS, 0.1% CHAPS and 2% BSA. At the end of the incubation time, cells were washed three times with PBS and incubated with either Alexa488-conjugated or Texas-Red-conjugated secondary antibodies and DAPI for 1 h at room temperature. For mitochondria labeling, neurons were incubated with 100 nM MitoTracker Red CMXRos™ dye (Invitrogen) in the culture medium for 45 min at 37°C, then fixed, permeabilized and labeled for phospho-Tyr216-GSK-3β as mentioned above. Finally, cells were washed three times with PBS and prepared for microscopy observation.

### Tetramethylrhodamine methyl ester (TMRM) labeling

Coverslips with cells were mounted in a non perfusion holder (37°C) placed on the stage of a laser-scanning confocal microscope (TCS SP5, Leica Microsystems, Rueil-Malmaison, France) equipped with a 63×/1,4 oil immersion objective. One minute after the beginning of each experiment, cells were loaded with the potentiometric dye TMRM (Sigma-Aldrich) in Krebs-Ringer at a final concentration of 30 nM at 37°C. TMRM was excited at 561 nm and its fluorescence was detected through a 570–600 nm spectral window. In order to follow the dye equilibration between cytosolic and mitochondrial compartments, images were collected every 10 sec for 10 min. At the end of the equilibration period, neuronal cultures were treated with 5 µM carbonyl cyanide *p*-(trifluoromethoxy)phenylhydrazone (FCCP) (Sigma-Aldrich) as a control for mitochondrial membrane depolarization. The resulting image stacks were processed using “*National Institutes of Health*” Image software™. The fluorescence intensity kinetic plots were obtained by selecting 8 regions of interests (roi) and expressed as the mean±S.D. of the mean gray values derived from all the roi selected in each image.

### Western blotting

Cells were rinsed with PBS and homogenized in lysis buffer (50 mM Tris-HCl, pH 7.5, 150 mM NaCl, 5 mM EDTA containing 0.5% Triton X-100 and 0.5% sodium deoxycholate). Equal amounts of total proteins (40 µg) determined by the Bradford method (BioRad, Marnes-La-Coquette, France) were separated onto 12% SDS-PAGE then transferred on nitrocellulose membrane (Schleicher & Schuell, Dassel, Germany). Blots were incubated in PBS 0.1% Tween, 1% milk with antibodies directed against GSK-3β (Santa Cruz Biotechnologies, Tebu-Bio, Le Perray-en-Yvelines, France), phospho-Tyr216-GSK-3β (BD Biosciences), phospho-Ser9-GSK-3β (Cell Signaling), cytochrome c (BD Biosciences), and then with the appropriate HRP-secondary antibody. Blots were developed using an enhanced chemoluminescence system (ECL, Amersham, GE Healthcare, Orsay, France) with a LAS3000 detector (Fuji). To correct for any loading artifact, same blots were re-probed with either anti-β-tubulin or anti-actin antibody (Abcam, Paris, France). Densitometry analyses were performed with a “*National Institutes of Health*” Image software™ on the immuno-positive bands. Results were expressed as a percentage of control load.

### Cell fractionation

Cells were lysed in buffer containing 20 mM Hepes, pH 7.5, 250 mM sucrose, 10 mM KCl, 1.5 mM MgCl2, 1 mM EGTA, 1 mM dithiothreitol and a protease inhibitor mix (Roche, Basel, Switzerland). Homogenates were centrifuged at 750×g for 10 min at 4°C, then supernatants were collected and centrifuged at 10 000×g for 15 min at 4°C. Resulting pellets were mitochondrial-enriched fractions and were washed twice in order to minimize putative contamination with cytosolic proteins. Supernatants were further centrifuged at 100 000×g for 1 h at 4°C, the resulting pellets were microsomal-enriched fractions while supernatants contained cytosolic proteins.

### Cell survival measurement

The 3-(4,5-dimethylthiazol-2-yl)-5-(3-carboxymethoxyphenyl)-2-(4-sulfophenyl)-2*H*-tetrazolium inner salt (MTS) assay (Promega, Charbonnières-les-Bains, France), a marker of mitochondrial activity, was done according to the manufacturer's recommendations. The percentage of cell survival was calculated by dividing the absorbance value of the MPP^+^-treated samples by that of the untreated control within each group.

### Flow cytometry analyses

MPP^+^- and untreated TSM1 neurons were pelleted and washed twice with PBS and the percentage of cells with active caspase-3 was assessed using PE-conjugated monoclonal active caspase-3 antibody kit (Pharmingen, BD Biosciences).

To measure the mitochondrial membrane potential, TSM1 cells were stained with fluorescent probe JC-1 (5,5′, 6, 6′-tetrachloro-1,1′,3,3′-tetraethylbenzimidazole carbocyanide iodide, BD Biosciences) at 1.0 µg/mL for 15 min at 37°C, washed and analyzed by flow cytometry. Photomultiplier settings were adjusted to detect green flurorescence of JC-1 monomers on FL-1 detector and the red fluorescence of JC-1 aggregates on the FL2 detector. Flow cytometry analysis was performed on a FACSCalibur™ and the CELLQuest™ software. Cells were gated according to size and scatter to eliminate dead cells and debris from analysis.

### Statistic analysis

Data were expressed as the mean±SD. Statistical analyses were performed using one-way analysis of variance (ANOVA) or Student *t*-test as indicated in the figure legends. *p* value<0.05 was considered as statistically significant.

## Results

### MPTP activated GSK-3β in dopaminergic neurons of mouse brain and in two neuronal cell models

*In vivo*, MPTP treatment is a well characterized and suitable animal model of PD pathogenesis. MPTP is known to induce selective death of dopaminergic neurons (i.e. Tyrosine Hydroxylase, TH-positive neurons) in the *substantia nigra pars compacta* (SNc) and to reproduce neuropathological features of PD in mice [35], [36].

**Figure 1:**
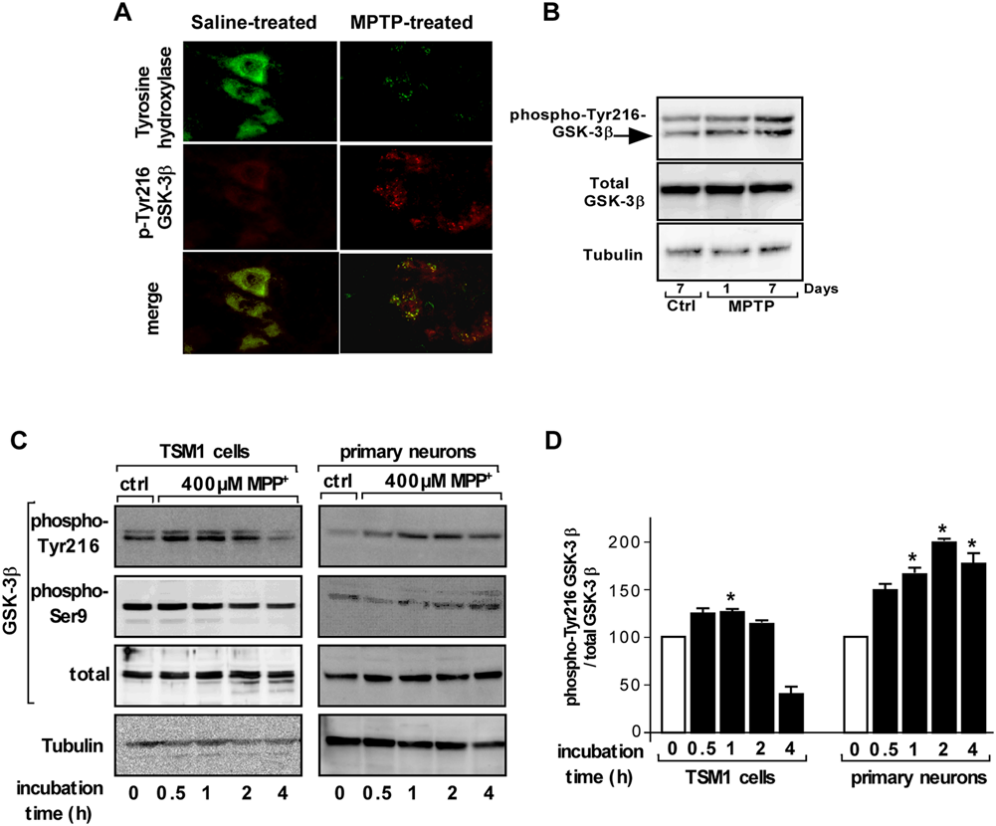
*In vivo* and *in cellulo* GSK-3β activation by MPTP/MPP^+^. (A) Seven days after the last saline or MPTP administration, coronal midbrain slices from saline- or MPTP-treated mice were labeled with anti-TH antibody (*green*) and anti-phospho-Tyr216-GSK-3β (*red*) as described in Materials and Methods. Merged micrographs illustrated the co-labeling (*yellow*) of anti-phospho-Tyr216-GSK-3β and anti-TH antibodies. Images are representative of three independent experiments. (B) Saline and MPTP-treated mice were killed one or seven days after the end of the MPTP administration. The brains were homogenized and equal protein amounts were analyzed by Western blotting for phospho-Tyr216-GSK-3β, total GSK-3β and β-tubulin contents. Arrowhead indicates the phospho-Tyr216-GSK-3β, the upper immuno-positive band is a non specific labeling. (C) TSM1 (*left*) and primary cultures of neurons (*right*) were incubated with saline (*ctrl*) or MPP^+^ (400 µM) for the indicated times (0.5, 1, 2 and 4 h). Total proteins (40 µg) were analyzed for phospho-Tyr216-GSK-3β, phospho-Ser9-GSK-3β, total GSK-3β and β-tubulin by Western blotting. (D) Quantification of blots from three independents experiments performed on TSM1 (*left*) and primary cultured neurons (*right*). The phospho-Tyr216-GSK-3β content in every condition was normalized to the total GSK-3β content and expressed as percentage of the control condition. The results are mean±SD and statistical analysis was done using Student *t*-test. *p<0.01 *versus* untreated sample.

In an attempt to study the role of endogenous GSK-3β *in vivo* in MPTP-induced dopaminergic neuronal cell death, we first examined the activation of GSK-3β (i.e phosphorylation at Tyr216 residue) in the SNc of MPTP-treated mice. Interestingly, immunohistochemistry analysis showed a selective activation of GSK-3β in TH-positive neurons evidenced by enhanced phosphorylation at Tyr 216 in remaining TH-positive neurons 7 days post MPTP-treatment as compared to saline-injected control brains (Figure 1A). To further confirm that the MPTP treatment induced GSK-3β activation, the phosphorylation state of Tyr216 residue of GSK-3β was also evaluated by Western blotting in mouse brain homogenates. The level of phospho-Tyr216-GSK-3β was significantly increased 7 days but not one day after the MPTP administration when compared to saline-treated controls (Figure 1B). In preliminary experiments, we established the kinetic of loss of TH-immunoreactive neurons *in vivo*. Mice were i.p. injected with 30 mg/kg every day for 5 consecutive days, then sacrificed 1, 3, 5 and 7 days after the last MPTP injection and brain sections were examined for TH immunoreactivity. Neuropathological examination of MPTP-treated mice revealed a progressive decrease in the number of TH-positive neurons in the SNc as early as 3 days after the MPTP treatment as compared to the saline-treated mouse brains. The maximal effect was observed 7 days after the last MPTP injection (data not shown).

MPP^+^ has been extensively used as an *in vitro* model of PD [37]. Based on preliminary results, we systematically used, in the following experiments with neuronal cells, a concentration of MPP^+^ leading to a significant cellular toxicity (i.e. 400 µM). Immortalized murine cortical neurons (TSM1) or primary cultures of neuronal cells were incubated in the presence of MPP^+^ iodide then Western blot analyses were performed on neuron homogenates in order to examine the effect of MPP^+^ on the phosphorylation of GSK-3β at Tyr216 (i.e. activated GSK-3β) and Ser9 residues (i.e. inactivated GSK-3β) (Figure 1C). MPP^+^ rapidly increased the phosphorylation of GSK-3β at residue Tyr216 in both neuronal cell types reaching a maximal effect after 1 and 2 h of incubation time for TSM1 and primary cultured neurons respectively as compared to the saline-treated cells (Figure 1C and 1D). Quantification after standardization of the same blots using the anti-β-tubulin and anti-total GSK-3β antibodies indicated a maximal effect after 1 and 2 h of MPP^+^ treatment in TSM1 cells and neurons in primary cultures respectively (126.5±2.3% and 180.1±4.5% compared to the control conditions taken as 100%) (Figure 1D). Surprisingly, the MPP^+^-induced GSK-3β phosphorylation at Tyr216 was persistent over 4 h in primary cultured neurons whereas it was transient in TSM1. The level of GSK-3β phosphorylation at residue Ser9 was less affected by MPP^+^ treatment than that of Tyr216. However, a slight increase of Ser9 phosphorylation was observed after 2 h of MPP^+^ treatment in primary cultures of neuronal cells (Figure 1C).

### Pharmacological and siRNA-mediated down-regulation of GSK-3β activity reduced MPP^+^-induced neuronal cell death

**Figure 2:**
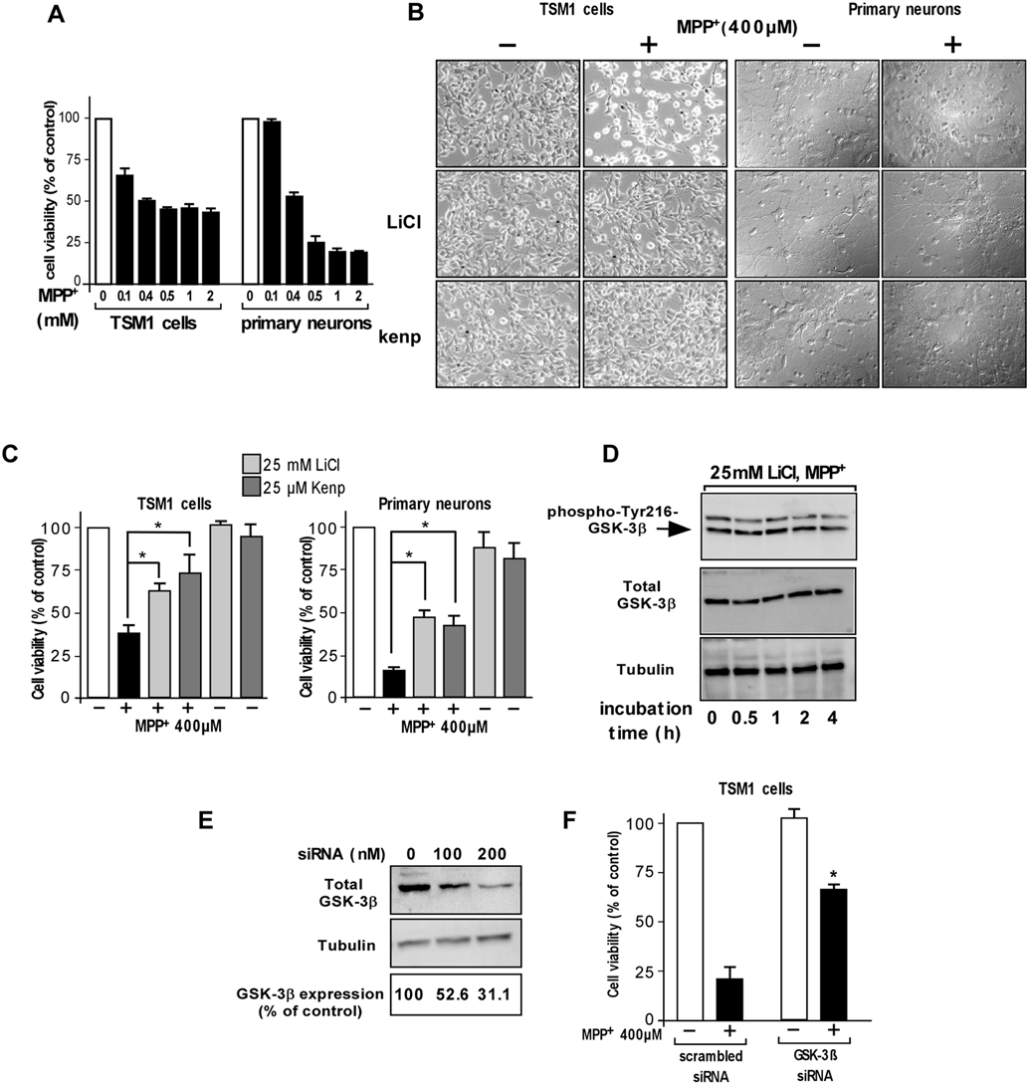
Effect of the down-regulation of GSK-3β activity on MPP^+^-induced neuronal cell death. (A) TSM1 and primary cultured neurons were treated with MPP^+^ (from 0.1 to 2 mM) for 15 h and cell viability was monitored by MTS assay. The data are expressed as the percentage of viable neurons when compared to untreated neurons and are the mean of three independent experiments with triplicate determinations ±S.D. (B) Phase-contrast pictures of representative microscopic fields of TSM1 (*left*) and primary cultured neurons (*right*) pre-treated with 25 mM LiCl or 25 µM kenpaullone for 15 min, and then treated with saline or MPP^+^ for additional 15 h at 37°C. Magnification, ×10. (C) TSM1 neurons (*left*) and mouse primary neurons (*right*) were pre-treated with LiCl or kenpaullone (25 mM and 25 µM, respectively), then incubated with MPP^+^ (400 µM) for 15 h at 37°C and cell viability was assessed using the MTS assay. The data are expressed as the percentage of viable neurons when compared to untreated neurons and are the mean of nine independent experiments with triplicate samples ±S.D. One-way ANOVA *p<0.01 *versus* saline-treated cells. (D) TSM1 neurons were pre-treated with LiCl (25 mM) for 15 min and co-incubated with 400 µM MPP^+^ for the indicated times (0.5, 1, 2 and 4 h) and total proteins were extracted and anaylzed for phospho-Tyr216-GSK-3β, total GSK-3β and β-tubulin contents by Western blotting. (E, F) TSM1 cells were transfected with scrambled siRNA (200 nM) or GSK-3β specific siRNA (100 or 200 nM) as described in Materials and Methods. Two days post-transfection, cells lysates were analyzed for total GSK-3β immunoreactivity (*upper panel*) by Western blotting (F). To determine the correction factor of load, blots were reprobed with the anti-β-tubulin antibody (*middle panel*). The expression level of GSK-3β was estimated by densitometry analyses performed with a “*National Institutes of Health*” Image software and expressed as the percent of control conditions. (E) Alternatively, scrambled- or GSK-3β specific- siRNA-transfected cells were treated with 400 µM MPP^+^ for 15 h at 37°C and the cell viability was monitored using MTS assay. Survival rate in every group was normalized to the untreated control cells. Error bars represent the S.D. for three independent experiments. Statistically significant differences were obtained between siRNA specific GSK-3β- and scrambled siRNA-transfected cells treated with 400 µM MPP+ using Student *t*-test (*p<0.05).

TSM1 and primary cultures of neuronal cells were incubated in the presence of increasing concentrations of MPP^+^ iodide (from 0.1 to 2 mM) for 15 h and the cell viability was evaluated using the colorimetric MTS assay (Figure 2A). In both TSM1 and primary neurons, MPP^+^ induced neuronal cell death in a concentration dependent manner. In both cell types, 400 µM MPP+ induced ∼50% of cell death (Figure 2A).

Lithium chloride (LiCl), a selective inhibitor of GSK-3β [38], was used to determine the implication of GSK-3β in MPP^+^-induced neuronal cell death. TSM1 and primary cultured neurons were incubated for 15 h with 400 µM MPP^+^ in the presence of GSK-3β inhibitors i.e. kenpaullone [39] and LiCl. Microscopy observations showed that the MPP^+^ treatment induced a significant decrease in the number of TSM1 cells and primary neurons as compared to the control condition (Figure 2B). Moreover, neurons exposed to MPP^+^ were dramatically damaged as reflected by the disappearance of normal cell bodies and the presence of shortened neuritis compared to untreated control cells (Figure 2B). The co-incubation with GSK-3β inhibitors kenpaullone or LiCl prevented MPP^+^-induced neurotoxicity (Figure 2B). Neuronal viability was monitored by measuring the reduction of mitochondrial activity using the MTS assay. Preliminary experiments allowed us to show that the optimal protective effect of LiCl and kenpaullone on MPP^+^-induced cell death was obtained at 25 mM and 25 µM, respectively (data not shown). In TSM1 and primary cultures of neuronal cells, LiCl or kenpaullone significantly reduced the MPP^+^-induced toxicity (Figure 2C). Treatment of TSM1 cells and primary neurons with 400 µM MPP^+^ decreased by ∼60% and ∼80% the cell viability as compared to the control, respectively. Co-treatment with 25 mM LiCl or 25 µM kenpaullone significantly protected TSM1 from the MPP^+^-induced death (Figure 2C). No significant toxicity was found when neurons were incubated with 25 mM LiCl or 25 µM kenpaullone alone (Figure 2B and 2C).

In an attempt to check the effect of LiCl on MPP^+^-induced GSK-3β activation, TSM1 cells were pre-treated with 25 mM LiCl for 15 min and then co-incubated with 400 µM MPP^+^ for various incubation times. Western blot analyses using anti-phospho-Tyr216-GSK-3β specific antibodies showed that LiCl prevented the MPP^+^-induced phosphorylation of GSK-3β at Tyr216 residue suggesting that LiCl hampered the MPP^+^-induced activation of GSK-3β (Figure 2D).

We used RNA interference, a gene silencing method, to further study the role of GSK-3β in MPP^+^-induced neuronal cell death. GSK-3β specific or control siRNA were transfected into TSM1 neurons and the level of GSK-3β was estimated by Western blot analysis. 200 nM of specific GSK-3β siRNA induced a ∼70% decrease of the expression level of GSK-3β (Figure 2E). siRNA transfected cells were then treated with MPP^+^ for an additional 15 h and the cell viability was monitored. The survival of GSK-3β siRNA-transfected cells was about 40% higher than that of the scrambled siRNA-transfected cells following MPP^+^ treatment indicating that MPP^+^-induced neurotoxicity was at least partially dependent on the expression of GSK-3β (Figure 2F).

### GSK-3β inhibitors prevented MPP^+^-induced caspase-9 and caspase-3 activation

**Figure 3:**
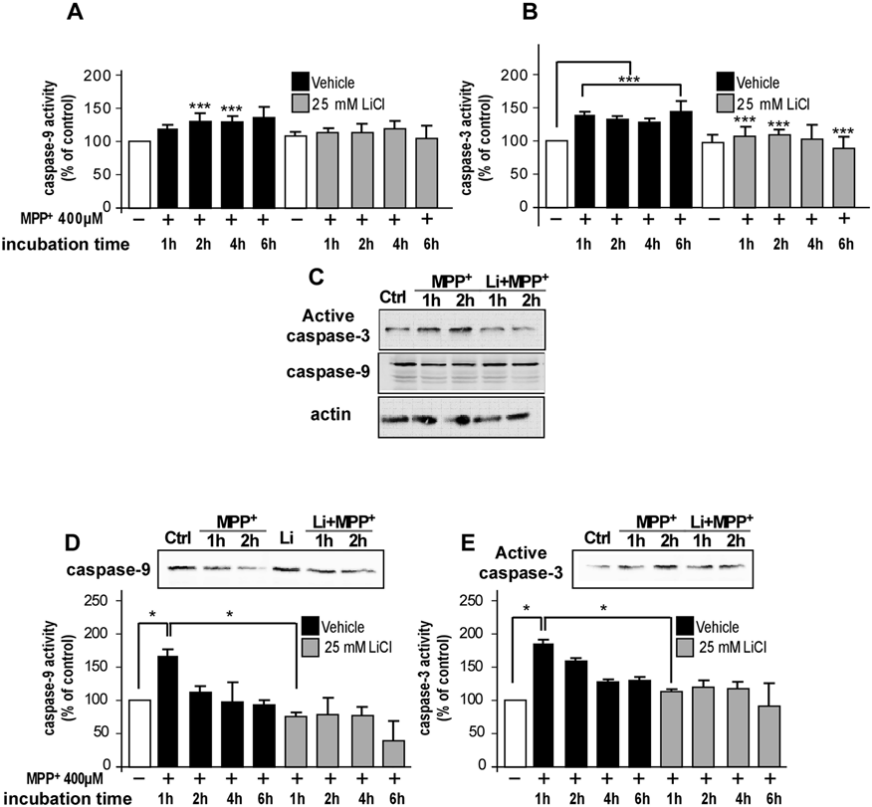
GSK-3β inhibitors prevented MPP^+^-induced caspases-9 and -3 activation. (A, B, C) TSM1 neurons were pre-treated with saline or 25 mM LiCl and then incubated with 400 µM MPP^+^ for indicated times (1, 2, 4, or 6 h). Intracellular caspase-9 (A) and caspase-3 (B) activities were measured by fluorescence enzymatic assay in cell homogenates. The data are expressed as the percentage of control when compared to untreated neurons and are the mean of three independent experiments with triplicate samples ±S.D. Statistical analysis was done using Student *t*-test (***p<0.005 *versus* untreated sample). (C) TSM1 cells were pre-treated or not with 25 mM LiCl and incubated with saline (*ctrl*) or MPP^+^ (400 µM) for 1 or 2 h. Total proteins (40 µg) were analyzed for active caspase-3, total caspase-9 and actin contents by Western blotting. (D, E) Primary cultured neurons were pre-treated with saline or 25 mM LiCl and then incubated with 400 µM MPP^+^ for indicated times (1, 2, 4, or 6 h). Intracellular caspase-9 (D) and caspase-3 (E) activities were measured by fluorescence enzymatic assay in cell homogenates. The data are expressed as the percentage of control when compared to untreated neurons and are the mean of three independent experiments with triplicate samples ±S.D. Statistical analysis was done using Student *t*-test (*p<0.05 *versus* untreated sample). Total caspase-9 (D) and active caspase-3 (E) contents were analyzed by Western blotting of primary neurons pre-treated or not with 25 mM LiCl and incubated with saline (*ctrl*) or MPP^+^ (400 µM) for 1 or 2 h.

To further characterize the intracellular pathways involved in the cell death induced by MPP^+^, we examined by fluorimetric assays and Western blot the activation of caspase-9 and caspase-3 after incubation with MPP^+^ in the presence or in the absence of pre-treatment with LiCl and kenpaullone (Figure 3). The MPP^+^ treatment induced a significant activation of caspase-9 in both neuronal models as compared to the untreated control, the maximal significant effect being observed after a 2 h-treatment for TSM1 cells and a 1 h-treatment for primary neurons (129.37±11.61% and 165.80±10.92% compared to the control conditions taken as 100%, Figure 3A and 3D). Significant caspase-3 activation was also observed in both cell types after as soon as 1 h after MPP+ treatment, reaching 138.84±5.44% of the control in TSM1 cells and 182.26±6.10% of the control in primary neurons (Figure 3B and 3E). Western blot analysis confirmed the increase of active caspase-3 immunoreactivity and the concomitant decrease of total caspase-9 level in TSM1 (Figure 3C) and primary cultured neurons (Figure 3D and 3E) upon MPP^+^ treatment. As shown by fluorimetric and Western blot analysis, pre-incubation with 25 mM LiCl prevented the MPP^+^-induced caspase-9 and -3 activation (Figure 3A–3E). Similar results were found by flow cytometry analysis of activated caspase-3 after incubation with MPP^+^ in the presence or in the absence of pre-treatment with LiCl and kenpaullone (data not shown). The MPP^+^ treatment induced the activation of caspase-3 in TSM1 neurons up to 233.9% as compared to the untreated control of active caspase-3 content of TSM1 cells. Pre-incubation with LiCl or kenpaullone minimized the MPP^+^-induced caspase-3 activation (194.9% and 198.3%, respectively).

### MPP^+^ differentially affected GSK-3β phosphorylation at residue Tyr216 in mitochondrial and cytosolic fractions

**Figure 4:**
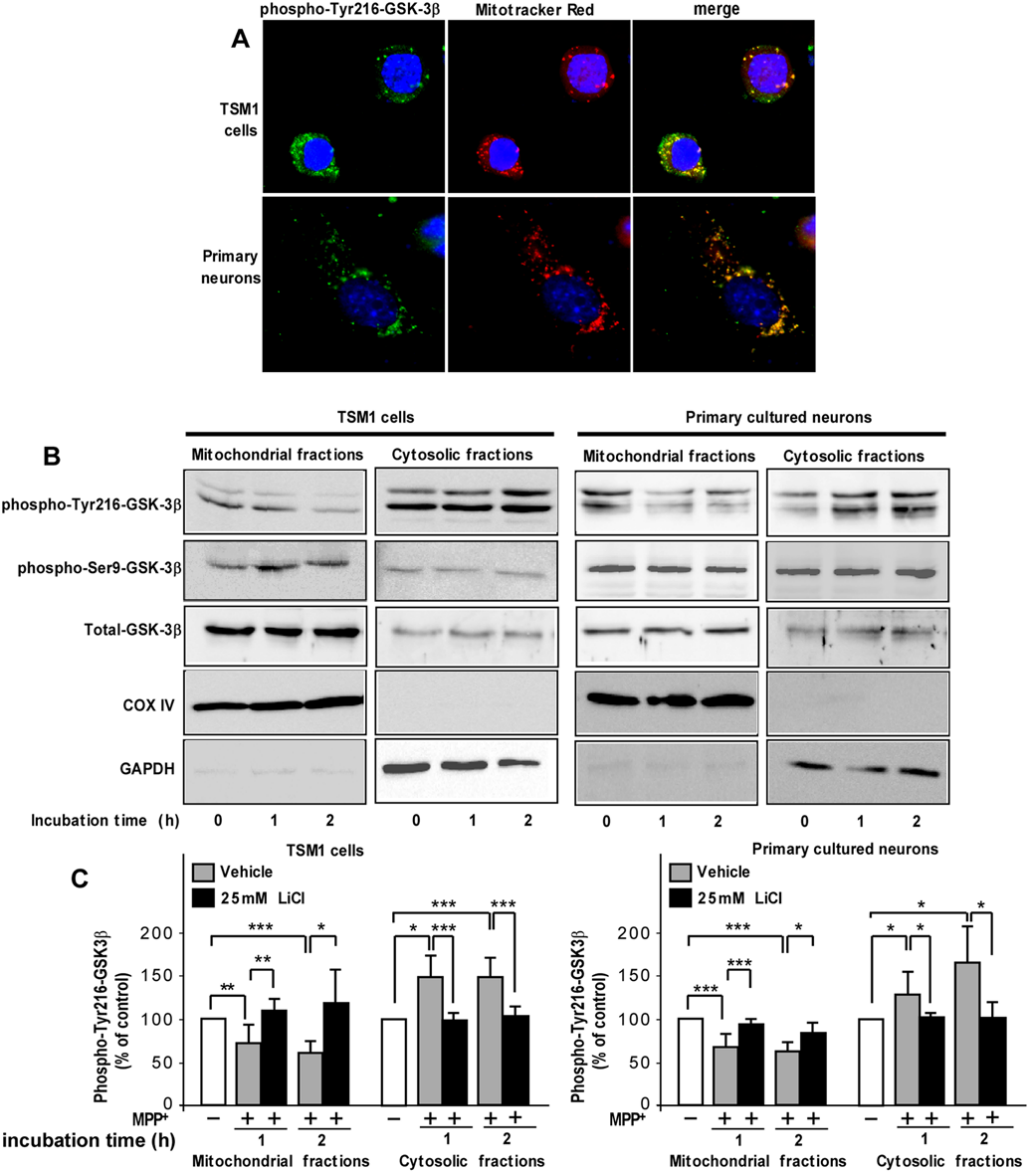
GSK-3β partially localized within mitochondria and MPP^+^ differentially affected phospho-Tyr216-GSK-3β in mitochondrial and cytosolic fractions. (A) Fluorescence microscopy pictures of TSM1 and primary cultures of neurons loaded with 100 nM MitoTracker™ (*red*), then fixed, permeabilized and stained for phospho-Tyr216-GSK-3β (*green*) and nuclei (DAPI, *blue*). Magnification ×25. (B, C) TSM1 neurons (*left*) and cultured primary neurons (*right*) were treated with 400 µM MPP^+^ for 1 and 2 h then submitted to subcellular fractionation in order to separate mitochondrial and cytosolic fractions, as described in Materials and Methods. Both fractions were analyzed for phospho-Tyr216-GSK-3β, phospho-Ser9-GSK-3β, total GSK-3β, COXIV and GAPDH contents by Western blotting (B). (C) Densitometry analyses of four to eight independent experiments were carried out to quantify the phospho-Tyr216-GSK-3β content in mitochondrial and cytosolic cellular fractions of vehicle- and LiCl-pretreated TSM1 (*left*) and primary cultured neurons (*right*). Data are expressed as percentage of the untreated samples. Statistical analysis was done using Student *t*-test (* *p*<0.05, ** *p*<0.01, ***p<0.005 *versus* untreated sample).

Previous studies have shown that GSK-3β exerts its functions *via* the mitochondrial activity, by modulating transport through the outer membrane of the mitochondria, regulating the opening of the mPTP or mediating the access of Bax to the mitochondria [27], [31], [40]. Using immunocytochemistry we first checked that in our neuronal models GSK-3β was indeed detectable within mitochondria. To do so, TSM1 and primary cultured neurons were loaded with the MitoTracker™ dye, then stained with phospho-Tyr216-GSK-3β specific antibody. Microscopy observation revealed that in both neuronal models, phospho-Tyr216-GSK-3β partially localized within mitochondria as evidenced by the overlap between phospho-Tyr216-GSK-3β and MitoTracker™ labeling (Figure 4A). We then investigated the effect on MPP^+^ treatment on the subcellular distribution of active and inactive forms of GSK-3β in TSM1 and primary neurons. MPP^+^-treated or untreated cells were submitted to differential subcellular fractionation and extracted proteins from the cytosolic and mitochondrial compartments were analyzed by Western blot. As early as 1 h after the MPP^+^ treatment, there was a marked decrease in phospho-Tyr216-GSK-3β in the mitochondrial fractions of both cell types that was even more pronounced after 2 h of MPP^+^ incubation time (Figure 4B and 4C). On the opposite, the cytosolic fraction of phospho-Tyr216-GSK-3β was significantly increased after the MPP^+^ treatment (Figure 4B and 4C). The mitochondrial and cytoplasmic fractions of the inactive form of the kinase i.e. phospho-Ser9-GSK-3β were not affected by MPP^+^ treatment in both compartments (Figure 4B). No significant effect was shown on the subcellular distribution of total GSK-3β, suggesting that modifications of the level of active form of GSK-3β likely resulted from specific phosphorylation mechanisms and not from protein relocation or neosynthesize process. To monitor the purity of mitochondrial and cytosolic fractions, COXIV and GAPDH immunoreactivities were assessed as mitochondrial and cytosolic specific markers, respectively (Figure 4B). Interestingly, the modifications of phospho-Tyr216-GSK-3β in cytosolic and mitochondrial fractions upon MPP^+^ treatment were significantly reduced by LiCl pre-treatment in both cell types (Figure 4C).

### Pharmacological and siRNA-mediated down-regulation of GSK-3β activity attenuated MPP^+^-induced decrease of mitochondrial membrane potential

**Figure 5:**
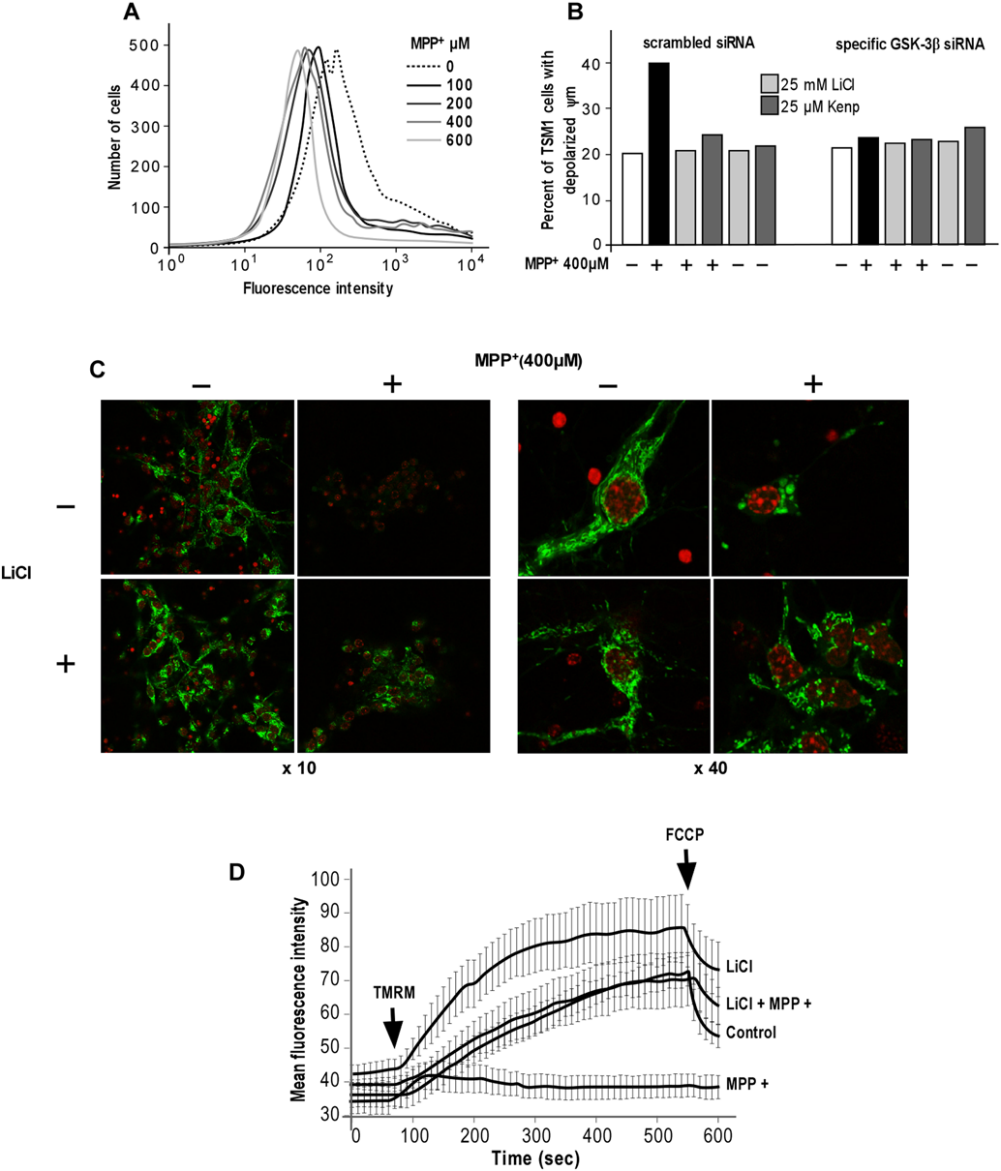
GSK-3β contributed to MPP^+^-induced TSM1 neuronal cell death through alterations of the mitochondrial membrane potential. (A) TSM1 neurons were treated with the indicated concentrations of MPP^+^ for 8 h then cells were stained with JC-1 and analyzed by flow cytometry. (B) TSM1 cells were cultured for 24 h then transfected with scrambled or GSK-3β-specific siRNA (200 nM). Two days post-transfection, cells were pre-treated with saline buffer or 25 mM LiCl or 25 µM kenpaullone (*kenp*), then co-incubated or not with MPP^+^ (400 µM) for 8 h. Cells were stained with JC-1 as described in Materials and Methods and prepared for flow cytometry analysis. The results are expressed as the percent of cells with depolarized mitochondrial membrane potential (Ψm). (C, D) intact primary neurons were pre-treated of not with 25 mM LiCl, then co-incubated or not with MPP+ (400 µM) for 4 h. Cells were placed on the stage on a laser-scanning confocal microscope and images were collected every 10 seconds for 10 minutes. One minute after the beginning of experiment, neurons were loaded with 30 nM TMRM (arrow) and one minute before the end of recording, 5 µM FCCP was added to the incubation medium (arrow). (C) Primary neurons pictures were collected 10 minutes after TMRM (*green*) and Hoechst dye (*red*) was added to the incubation medium. Magnification, ×63. (D) The fluorescence intensity plots were obtained by selecting eight roi and expressed as the mean±S.D. of the mean gray values derived from all the selected roi in each image.

One common feature of PD is the reduction of complex I activity in the SNc, supporting the hypothesis that nigral dopaminergic neurons are highly vulnerable to stress arising from mitochondrial dysfunction [41]. To investigate the involvement of mitochondria in MPP^+^-induced neuronal cell death through GSK-3β activation, we analyzed the changes of mitochondrial membrane potential after MPP^+^ treatment by flow cytometry analysis using the fluorescent probe JC-1. JC-1 is selectively taken up by the mitochondria and is a reliable indicator of change of the mitochondrial membrane potential (ΔΨm). We first studied the effects of a dose-response of MPP^+^ (from 100 to 600 µM) on mitochondrial membrane potential in TSM1 neurons (Figure 5A). Increasing concentrations of MPP^+^ induced a dose-dependent reduction of the fluorescence intensity as compared to that obtained with untreated cells, indicating a progressive MPP^+^-dependent depolarization of the mitochondrial membrane (Figure 5A). To investigate whether GSK-3β exerts its neurotoxic role by modifying the mitochondria membrane potential, scrambled or GSK-3β specific siRNA-transfected TSM1 cells were treated with MPP^+^ alone or in combination with LiCl or kenpaullone. The number of cells with depolarized mitochondrial membrane potential remained unchanged in GSK-3β specific siRNA-transfected cells whereas it was increased by a two-fold factor in scrambled siRNA-transfected cells upon MPP^+^ treatment (Figure 5B). As expected, pre-treatment with LiCl or kenpaullone totally abolished the effect of MPP^+^ on the mitochondrial membrane potential in scrambled siRNA-transfected cells. These results indicate that the expression of GSK3β is required for the MPP^+^-induced alterations of the mitochondrial membrane potential.

The fluorescent membrane-permeant cationic probe tetramethylrhodamine methyl ester (TMRM) has become one of the most frequently used probes in the analysis of variations of the mitochondrial membrane potential in intact cells because it exhibits very low toxicity and displays rapid and reversible membrane equilibration properties. We performed confocal microscopy analysis on intact primary cultured neurons in order to compare the kinetic and level of TMRM integration in mitochondria of control, MPP^+^ and LiCl/MPP^+^-treated neuronal cells. In control neurons, TMRM rapidly entered within mitochondria where it accumulated in an inner-membrane potential dependent manner (Figure 5C), as illustrated by the rapid increase of mean fluorescence intensity of untreated cells (Figure 5D). In MPP^+^-treated cells, TMRM only minimally entered within mitochondria and almost no TMRM fluorescence was detected (Figure 5C and 5D), indicating that MPP^+^ induced the depolarization of mitochondrial membrane potential. Interestingly, TMRM accumulated similarly in control cells and in LiCl/MPP^+^ co-treated neurons, suggesting that LiCl treatment prevented the MPP^+^-induced mitochondrial membrane depolarization (Figure 5C and 5D). LiCl treatment alone didn't affect mitochondria morphology and distribution (Figure 5C) and strongly increased TMRM mean fluorescence intensity during the course of the experiment, indicating that LiCl changed mitochondrial membrane potential (Figure 5D). FCCP, a mitochondrial uncoupler, was used as control because it induced a mitochondrial membrane potential collapse in intact cells. When FCCP was added to neurons, TMRM no longer accumulated inside mitochondria and became evenly distributed throughout the cytosol, as shown by the loss of TMRM fluorescence in control cells (Figure 5D).Taken together,these results indicated that GSK-3β triggered a strong depolarization of the mitochondrial membrane potential in neuronal cells exposed to MPP^+^.

## Discussion

In the present study, we examined the implication of GSK-3β in MPP^+^-induced mitochondrial dysfunction, and the putative protective role of GSK-3β inhibition on MPP^+^-mediated neurotoxicity.

The role of mitochondria in the pathogenesis of several neurodegenerative disorders, including PD, has been well documented. Consistent deficits in the subunits and activity of mitochondrial complex I of the electron transport chain in blood platelets and *substantia nigra* of PD patients are prominent phenomena [42], [43]. Reduced complex I activity is also seen in cytoplasmic hybrid cell lines containing DNA from PD patients [44]. The role of mitochondrial dysfunction in PD also extends beyond that of a respiratory chain defect since mutations in three nuclear genes (PINK1, DJ1 and Omi) encoding mitochondrial proteins have been described in familial forms of PD. However, little is known regarding the molecular mechanisms leading to mitochondrial dysfunction but reduced mitochondrial membrane potential might be considered as an initial and irreversible step towards apoptosis.

GSK-3β is a well-known determinant in apoptotic process leading to neuronal cell death in several paradigms. For instance, the involvement of GSK-3β in neuronal apoptosis has been demonstrated following excitotoxic treatment [45], staurosporine-induced cell death [46], β-amyloid and MPTP treatments [47], [48]. Activated GSK-3β, which inhibits heat shock transcription factor (HSTF)-1 and activates the mitochondrial death pathway, results in increased cytochrome c release from mitochondria. This, in turn, activates successively caspase-9 and caspase-3, accompanied by poly (ADP-ribose) polymerase (PARP) cleavage [46], [49], [50], [51]. Moreover, GSK-3β has been shown to be located into several cell compartments such as cytosol, nucleus and mitochondria by both microscopy and immunoblotting experiments [26], [52], [27], [28]. The translocation of GSK-3β from the cytosol to the nucleus is known to be one of the regulatory mechanisms of its function [14], [53]. However, the significance of the presence of GSK-3β in mitochondria is still completely misunderstood. GSK-3β may play crucial roles in the regulation of cell death and survival by modulating mitochondrial apoptotic cell death pathway [54], [55]. Interestingly, GSK-3β has also been described to be involved in the regulation of opening of the mPTP, an event that can occur under conditions of oxidative stress or electron transport chain inhibition, leading to collapse of the mitochondrial membrane potential [30], [56], [57].

The current study demonstrates several findings regarding the role of GSK-3β in MPP^+^-induced mitochondrial dysfunction and neuronal cell death. The best-characterized mechanism for the inactivation of GSK-3β occurs through the phosphorylation of its N-terminus at Ser9 by the kinase Akt [58]. However, in our study, we show that, *in vivo*, MPTP treatment induces a progressive loss of TH-positive neurons concomitant with an increase of phospho-Tyr216 GSK-3β in remaining dopaminergic neurons, indicating that the activation of GSK-3β by specific phosphorylation of Tyr216 may be involved in the MPP^+^-dependent neuronal cell death process. These *in vivo* results are in agreement with observations performed in immortalized TSM1 and primary cultured neurons. Indeed, in both neuronal cell types, the treatment with MPP^+^ induced an increase in the phosphorylation at Tyr216 of GSK-3β whereas only negligible decrease in the amount of phospho-Ser9 GSK-3β was observed. Thus, MPP^+^ does not appear to regulate the Akt-mediated phosphorylation of GSK-3β on Ser9, a result that differs from previous studies using different cell models [19], [22].

We demonstrated for the first time a cellular compartment-specifc regulation process of GSK-3β activity upon MPP^+^-treatment. Indeed, in MPP^+^-treated neurons, the active form of GSK-3β increases in the cytosol and concomitantly decreases in the mitochondrial fraction leading to a collapse of the mitochondrial membrane potential. The translocation of GSK-3β from the cytosol to mitochondria and its physical interaction with mitochondrial permeability transition pore complex has been previously suggested in an ischemia/reperfusion paradigm [57]. Despite the fact that the total GSK-3β amount and its distribution do not appear to be affected by MPP^+^ treatment, we cannot rule out the possibility of a translocation of the active form of GSK-3β from the mitochondria to the cytosol. Therefore, the active form of GSK-3β present in mitochondria could be determinant for the maintenance of the mitochondrial membrane potential. Consistent with this hypothesis, LiCl-treated neurons present higher mitochondrial membrane potential than untreated cells (Figure 4D). Moreover, we showed that the regulation of GSK-3β activity triggers MPP^+^-induced mitochondrial membrane depolarization, and subsequent mitochondrial-dependent activation of the caspase cascade. Taken together, our results suggest a dual role for active GSK-3β according to its subcellular location. MPP^+^ induces neuronal cell death through the mitochondrial dysfunction closely related to the down-regulation of mitochondrial GSK-3β activity and the up-regulation of active cytosolic form of the kinase.

Finally, to precisely evaluate the role of GSK-3β in PD-related cell death, we inhibited GSK-3β activity using two selective GSK-3β inhibitors, LiCl and kenpaullone, and gene silencing with a siRNA targeting GSK-3β. Lithium is widely used for the treatment of maniac depression [59], but it is also well known for its neuroprotective properties [60], [61]. Indeed, several reports have demonstrated that lithium, in neuronal cell models and *in vivo*, has neuroprotective activity *via* the up regulation of antiapoptotic and reduction of proapoptotic Bcl-2 family proteins, including Bcl-2 and Bax [21], [62]–[65]. Previous studies have demonstrated that GSK-3β contributes to the death of a wide variety of cells including SH-SY5Y, PC12 or cerebellar granule neurons [22], [24], [48], [66]–[68]. Moreover, the specific inhibition of GSK-3β could protect SH-SY5Y cells [20] and dopaminergic neurons against MPTP toxicity [19]. In this study, we show that LiCl blocked MPP^+^-induced phospho-Tyr216-GSK-3β in both TSM1 and primary cultured neurons and that LiCl and kenpaullone protected neurons from MPP^+^-induced neuronal phenotypic damages, neurotoxicity and apoptotic cell death.

GSK-3β is likely the major target of lithium to elicit neuroprotection, although other targets have also been described [69], [70]. However, we show that silencing the expression of GSK-3β by specific siRNA protected neurons against MPP^+^ toxicity and no synergistic neuroprotective effect of lithium can be observed, demonstrating that the neuroprotective effect of lithium actually involved GSK-3β inhibition.

In summary, we clearly demonstrated the central role of GSK-3β in MPTP/MPP^+^-mediated neuronal cell death and we showed that differential GSK-3β regulations take place within mitochondria and cytosol. Further investigations will be required to precisely determine the role of mitochondrial GSK-3β in physiological conditions. However, its activity may contribute to variation of the mitochondrial membrane potential. Our results highlight differential regulation of GSK-3β activity according to its subcellular compartimentalization. Indeed, inhibition of GSK-3β activity with specific inhibitors or siRNA significantly protected neurons from MPP^+^-induced cell death. New therapeutic strategy for PD targeting GSK-3β inhibition deserves to be evaluated keeping in mind the specific regulation of the different GSK-3β subcellular pools.
